# Gleaning evolutionary insights from the genome sequence of a probiotic yeast *Saccharomyces boulardii*

**DOI:** 10.1186/1757-4749-5-30

**Published:** 2013-10-22

**Authors:** Indu Khatri, Akil Akhtar, Kamaldeep Kaur, Rajul Tomar, Gandham Satyanarayana Prasad, Thirumalai Nallan Chakravarthy Ramya, Srikrishna Subramanian

**Affiliations:** 1 CSIR-Institute of Microbial Technology, Chandigarh, India

**Keywords:** Diarrhea, Human gut, *Clostridium difficile*, Enterohemorrhagic *Escherichia coli*

## Abstract

**Background:**

The yeast *Saccharomyces boulardii* is used worldwide as a probiotic to alleviate the effects of several gastrointestinal diseases and control antibiotics-associated diarrhea. While many studies report the probiotic effects of *S. boulardii*, no genome information for this yeast is currently available in the public domain.

**Results:**

We report the 11.4 Mbp draft genome of this probiotic yeast. The draft genome was obtained by assembling Roche 454 FLX + shotgun data into 194 contigs with an N50 of 251 Kbp. We compare our draft genome with all other *Saccharomyces cerevisiae* genomes.

**Conclusions:**

Our analysis confirms the close similarity of *S. boulardii* to *S. cerevisiae* strains and provides a framework to understand the probiotic effects of this yeast, which exhibits unique physiological and metabolic properties.

## Background

Probiotics are live microbes that assist in restoring the symbiotic intestinal gut flora balance and thus bestow health benefits to the host [1,2]. The most commonly used human probiotics are members of the *Lactobacillus* and *Bifidobacterium* species [3]. Besides these bacteria, *Saccharomyces boulardii* (*Sb*), a yeast strain, is also widely used as a probiotic to treat a variety of conditions [4] including antibiotics-associated diarrhea and recurrent *Clostridium difficile* infection. A primary advantage of using *Sb* as a probiotic is that it can be used by patients undergoing antibiotic regimen due to its natural resistance to antibiotics [5]. The genetic transfer of antibiotic resistance genes, a frequent event between pathogenic and gastrointestinal tract (GIT) bacteria, is not as frequent between yeast and bacteria [6,7]. Furthermore, *Sb* is also tolerant to various local stresses such as the presence of gastrointestinal (GI) enzymes, bile salts, organic acids *etc.* and can withstand considerable variations in pH and temperature while transiting through the human GIT [8].

*Sb* is a tropical strain of yeast that is thermophilic and mostly non-pathogenic to humans [5,9-12]. It was first isolated from the skin of lychee and mangosteen fruits in 1923 by the French scientist Henri Boulard in the Indo-China region, and has since then shown to be effective as a preventive and therapeutic agent for diarrhea and other GI disorders caused by the administration of antimicrobial agents [13]. The detailed and precise mechanisms of the action of *Sb* to confer protection against several diseases remain as yet unexplored although some specific proteins have been suggested to play key roles in its probiotic function. For example, a 54 kDa serine protease from *Sb* was reported to provide protection against *C. difficile* infections by cleaving toxins A and B [14,15]. Similarly, a 120 kDa protein has been suggested to play a role in neutralizing the secretion induced by the cholera toxin by reducing cyclic Adenosine Monophosphate (cAMP) levels [16]. *Sb* can also inhibit the *Escherichia coli* endotoxin by dephosphorylation mediated by a 63 kDa protein phosphatase [17]. Likewise, *Sb* can decrease IL-8 proinflammatory cytokine secretion in Enterohemorrhagic *E. coli* (EHEC) infections by inhibiting the NF-κB and Mitogen-Activated Protein Kinase (MAPK) signaling pathways [18]. *Sb* has also been shown to inhibit *Candida albicans* translocation from GIT of mice [19] and can inhibit *in vitro* adhesion of *Entamoeba histolytica* trophozoites to human erythrocytes [20] and *C. difficile* adhesion to vero cells [21]. These enteropathogens adhere to the host tissue surface as the initial event for infecting the host. The outer membrane of *Sb* which is particularly rich in mannose, as compared to other yeasts, adheres to the enteropathogens strongly and inhibits their binding to the mucous membrane of the GIT [22].

The taxonomic position of *Sb* has been an issue of intense debate [8]. *Sb* was initially suggested to be a new species of the hemiascomycota genus *Saccharomyces*[23]. Based on comparative electrophoretic karyotyping and multivariate analysis of the polymorphism observed in pulsed-field gel electrophoresis Cardinali and Martini [24] classified *Sb* outside of the *S. cerevisiae* group. However, molecular phylogenetics and typing using molecular techniques *viz.*, species-specific polymerase chain reaction (PCR), randomly amplified polymorphic DNA-PCR, restriction fragment length polymorphism analysis (RFLP) of rDNA spacer region and pulsed field gel electrophoresis (PFGE) helped identify *Sb* as a strain of *S. cerevisiae*[25]. Moreover, comparative genomic hybridization also established that *S. cerevisiae* and *Sb* are members of the same species [25]. But, *Sb* differs from other *S. cerevisiae* genetically as comparative genome hybridization using oligonucleotide-based microarrays reveal trisomy of chromosome IX and altered copy number of individual genes [26]. When compared to *S. cerevisiae* strains S288c and 1171 T there is 100% similarity in the D1/D2 domain sequence of the 26S rDNA of eight *Sb* strains and more than 95% similarity to mitochondrial cytochrome-c oxidase II gene (COX2) sequences [27]. Another differentiating criteria reported in literature is that *Sb* is incapable of metabolizing galactose as a source of carbon [23,28]. However, McCullogh et al. (1998) [29] have shown that galactose could be metabolized by some *Sb* strains. Therefore, we determined the genome sequence of *Sb* in order to get insights into the evolutionary history and taxonomic position and to get a better understanding of the various probiotic effects of this yeast, which exhibits unique physiological and metabolic properties.

## Methods

### Isolation and purification of *S. boulardii* genomic DNA

A sachet of Dr. Reddy’s Laboratories Econorm 250 mg (B. No. 1500, Mfg. Date: 05/12, Expiry Date: 04/14) containing lyophilized cells of *Sb* was used as the source of the probiotic yeast. Yeast cells were suspended in Milli-Q water, serially diluted, and plated on Yeast Mold (YM) agar (Difco) plates. The plates were incubated at 37°C for 48 hours. An isolated colony was picked from the plate and cultured in Yeast Extract-Peptone-Dextrose (YEPD) broth (HIMEDIA) for 24 hours at 37°C in a rotary shaker (180 RPM). The cells were centrifuged at 5000 g for 10 minutes and washed with distilled water. DNA isolation was performed using the ZR Fungal/Bacterial DNA mini prep kit (Zymogen) as per instructions in its user manual. After isolation, the genomic DNA was treated with RNase A (1 μl of a 10 μg/mL stock solution for 100 μl of solution containing DNA) and incubated at 37°C for 30 minutes. Then, 1/10 volume of 3 M sodium acetate (pH 5.2) and 2.5 volumes of absolute ethanol was added followed by incubation at −20°C overnight and centrifugation at 14,000 rpm for 30 minutes at 4°C. Supernatant was carefully discarded; pellet was rinsed with 70% ethanol and centrifuged again at 14,000 rpm for 15 minutes at 4°C. The ratio of OD at 260/280 nm was ~1.8 as observed by NanoDropND-1000 spectrophotometer.

### Internal transcribed spacer-polymerase chain reaction (ITS-PCR)

To amplify the ITS regions, primers ITS1 (TCCGTAGGTGAACCTGCGG) and ITS4 (TCCTCCGCTTATTGATATG) were used. Amplification was done using a mixture containing 1× Standard *Taq* Reaction Buffer, dNTPs (200 μM), BSA (0.3 μg/μL), template DNA (500 ng), 1.25 units/50 μl PCR *Taq* DNA Polymerase (Taq DNA Polymerase with Standard Taq Buffer; New England BioLabs) and forward and reverse primers (0.2 μM each). The cycling parameters used for amplification were: initial denaturation for 5 minutes at 95°C, followed by 30 cycles of 30 seconds at 95°C, 30 seconds at 50°C and 90 seconds at 72°C, with a final extension for 10 minutes at 72°C and cooling to 4°C. The amplified products were separated on 1.2% agarose gels, visualized and photographed on an Alpha Image Analyzer (Alpha Innotech Corporation, CA). The DNA from the amplified bands was eluted with QIAquick Gel Extraction Kit (Qiagen N.V.). The eluted DNA was further amplified for sequencing using Terminator Ready Reaction Mix (1 μl), Sequencing Buffer (1 μl; 5×; 200 mM Tris-Cl, 5 mM MgCl_2_, pH 9.0), PCR amplified DNA (35 ng), primer (3.2 pmol) and Milli-Q water to make up the volume to 10 μl. PCR cycling conditions were: initial denaturation for 1 minute at 96°C, followed by 24 cycles of 10 seconds at 96°C, 5 seconds at 50°C and 4 minutes at 60°C and cooling to 4°C. The final PCR product was sequenced on a Sanger sequencer and the resulting ITS sequence was compared against all available ITS sequences on the NCBI database.

### Microsatellite fingerprinting

Microsatellite fingerprinting based on the Post Meiotic Segregation (PMS) marker [30], PMS1, PMS2 and PMS3 was performed. Amplification was achieved using a mixture containing 1× Standard *Taq* Reaction Buffer, dNTPs (200 μM), BSA (0.3 μg/μL), template DNA (500 ng), 1.25 units/50 μl PCR *Taq* DNA Polymerase (Taq DNA Polymerase with Standard *Taq* Buffer; New England BioLabs) and 0.2 μM of forward and reverse primers (PMS1: GTGGTGGTGGTGGTG; PMS2: GACGACGACGACGAC and PMS3: GACAGACAGACAGACA). The following cycling parameters were used for microsatellite fingerprinting: initial denaturation for 5 minutes at 94°C, followed by 40 cycles of 1 minute at 94°C, 2 minutes at 45°C and 3 minutes at 72°C, with a final extension for 10 minutes at 72°C and cooling to 4°C. The amplified products were separated on 1.2% agarose gels, visualized and photographed on an Alpha Image Analyzer (Alpha Innotech Corporation, CA).

### Genome sequencing

*Sb* EDRL (Econorm - Dr. Reddy’s Laboratories) was sequenced using the 454/Roche GS FLX Titanium system. Library preparation was carried out according to the GS FLX Titanium Rapid Library Preparation Kit (Roche Applied Sciences) at Centre for Cellular and Molecular Platforms (C-CAMP), Bangalore, India. The genomic DNA was first sheared into 400–1000 base-pair-long fragments and its quality was assessed using a BioAnalyzer DNA 7500 LabChip. Blunt ends were generated and adaptor ligation was followed by removal of small fragments. After library immobilization, the library was quantified using the RiboGreen method and the final yield was calculated. Half PicoTiter Plate of 454 shotgun sequencing was performed and resulted in a total of 733,390 reads for *Sb* EDRL with ~50× coverage.

### Assembly, mapping and annotation

The 454 shotgun reads were assembled *de novo* using Newbler v2 [31]. Multiple assemblies were obtained by varying the parameters for the minimum overlap length (ml) and minimum overlap identity (mi). The assembly that contained the lowest number of contigs and best N50 score (parameters: minlen 45; mi 96; ml 100) was chosen for further analysis. The quality of the assembly was further checked by mapping back the reads on the draft genome and visually checking for errors. Feature annotation was carried out by the MAKER pipeline [32] and tRNA was predicted by tRNAscan-SE 1.23 [33]. Features thus annotated were subjected to BLASTp [34] for functional characterization of the proteins with an E-value cutoff of 1e^-5^. In addition to *de novo* assembly, the 454 shotgun reads were mapped onto 34 draft and complete genomes of *S. cerevisiae* available at NCBI (Additional file 1**)** using the mapping algorithm of CLCbio Genomics wb6 (http://www.clcbio.com).

### Comparative genomics

The draft genome annotated by the MAKER [32] pipeline and the reference-mapped genomes were searched for the presence of proteins in the molecular weight range of 54 kDa and 120 kDa using an in-house Practical Extraction and Report Language (PERL) script. The 63 kDa protein was retrieved by BLASTp using as query a 38-amino acid sequence stretch of this protein previously reported in literature [17], and the molecular weight was further confirmed using our PERL script. In order to narrow down on the 54 kDa protease, proteins in the molecular weight range 50–60 kDa, retrieved as mentioned above, were subject to BLASTp against the MEROPS database (http://merops.sanger.ac.uk/) and an independent hmmscan [35] run against the protein family (PFAM) database [36] with an E-value cutoff of 1e^-5^. These proteins were further used as queries in the Fold and Function Assignment System (FFAS) [37] program to retrieve annotated homologs and subjected to BLASTp against the Gene Ontology (GO) database [38] with an E-value cutoff of 1e^-5^. Likewise, the sequences of 119–121 kDa proteins obtained using our PERL script was subjected to BLASTp against the GO database with an E-value cutoff of 1e^-5^ and Search Tool for the Retrieval of Interacting Genes/Proteins (STRING) [39] analysis was performed to find interacting partners. Galactose metabolizing enzymes of the Leloir pathway [40] were found in the annotated genome by initiating stand-alone BLASTp searches using sequences of these enzymes from other *S. cerevisiae*.

### Quality assurance

**Figure 1:**
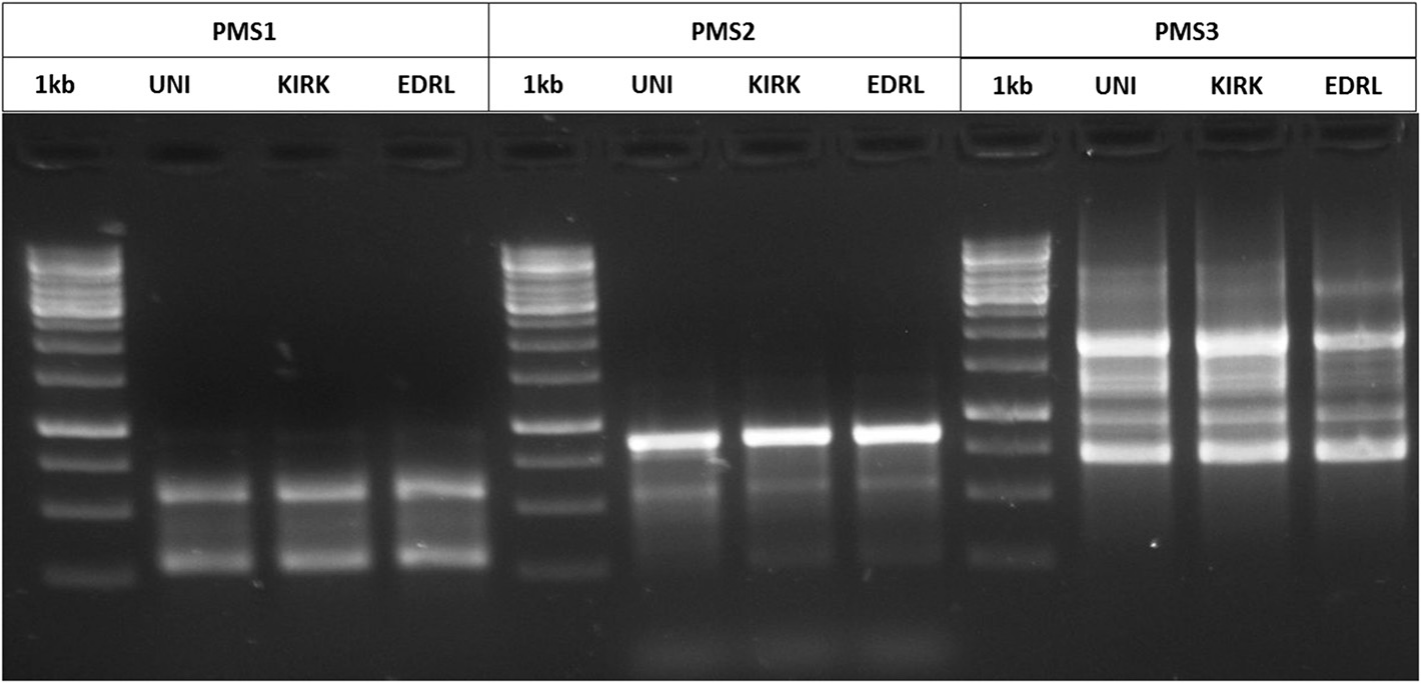
**Microsatellite fingerprinting of *Sb*: Microsatellite fingerprinting pattern for PMS molecular marker PMS1, PMS2 and PMS3; where UNI - *Sb *Uni-Sankyo Ltd.** (Now Sanzyme Ltd.); **KIRK **- *Sb* Kirkman; **EDRL **- *Sb* EDRL; and **M**- 1 kb DNA ladder.

The genomic DNA was purified from a commercially available lyophilized *Sb* (Econorm sachet; Dr. Reddy’s Laboratories) and was further confirmed by ITS sequencing. The ITS sequence was >99% identical to that of the *S. boulardii* culture collection (KC2540481.1) strain. PMS1, PMS2 and PMS3 markers were used to perform microsatellite fingerprinting in order to verify the similarity between *Sb* EDRL to other commercially available *Sb* strains (*Sb* Uni-Sankyo Ltd. [Now known as Sanzyme Ltd.] and *Sb* Kirkman) (Figure 1).

## Results and discussion

### Genome characteristics

Next-generation sequencing of *Sb* EDRL on the Roche 454 GS-FLX Titanium platform resulted in a total of 733,390 shotgun reads of length 40–1773 bp. High-quality reads with ~50× coverage were assembled using Newbler v2.8 to obtain a draft genome of 11.4 Mbp in 194 contigs (N50: 251,807 bp). The GC content was 38% and 285 tRNA were present in *Sb* EDRL. Feature annotation was performed using the MAKER pipeline with Augustus [41] as the gene predictor. Of the 5803 coding sequences (CDS) regions predicted, 4635 (79%) could find hits with *S. cerevisiae* proteins when subject to a BLAST analysis against the non-redundant (nr) database. Reference-based mapping of *Sb* data onto other *S. cerevisiae* genomes revealed that the maximum number of reads map to *S. cerevisiae* strains Lalvin QA23 followed by EC1118, RM11-1a and S288c suggesting high similarity to these genomes (Additional file 2).

### 54-kDa serine protease and 120 kDa protein

*Sb* is being used in treatment of *C. difficile*-induced diarrhea and colitis [14]. *Sb* can inhibit the toxins A and B of *C. difficile* by producing a 54 kDa serine protease that cleaves these toxins [14,15]. Approximately 600 proteins of the *Sb* genome in the molecular weight range of 50–60 kDa were subjected to BLASTp against the MEROPS database and of these, 221 hits were obtained. These proteins were further subjected to BLASTp runs against the GO database [38]. Four proteins were found to be putative serine proteases based on their GO annotation and presence of conserved serine protease signature motifs. These four proteins belong to the carboxypeptidase and subtilisin-like sub-classes of serine protease (Additional file 2 and Additional file 3). Independently, all the annotated proteins from the MAKER pipeline were subjected to hmmscan against the PFAM database. Twenty two serine proteases spanning 10 sub-classes of the serine protease family were obtained. Of these 22 proteins, 4 were in the molecular weight range of 50–60 kDa and were the same proteins identified by our previous search against the MEROPS database as putative serine proteases. It is therefore tempting to speculate that one or more of these four proteins could possibly play a role in cleaving the *C. difficile* toxins A and B*.*

A 120 kDa protein has been suggested to neutralize the effects of cholera toxin by reducing cAMP levels in mouse [17]. Fifteen proteins in the molecular weight range of 119–121 kDa were retrieved (Additional file 3). These proteins were subjected to BLASTp against the GO database [38] with an E-value cutoff of 1e^-5^ and FFAS search was performed. These 15 proteins mostly belong to the family of kinases and transporters (Additional file 2). The interacting partners of these proteins were fetched out by STRING analysis (Additional file 2). It is possible that any of these proteins may be involved in the cAMP pathway to neutralize the effects of the cholera toxin.

### 63-kDa phosphatase

**Figure 2:**
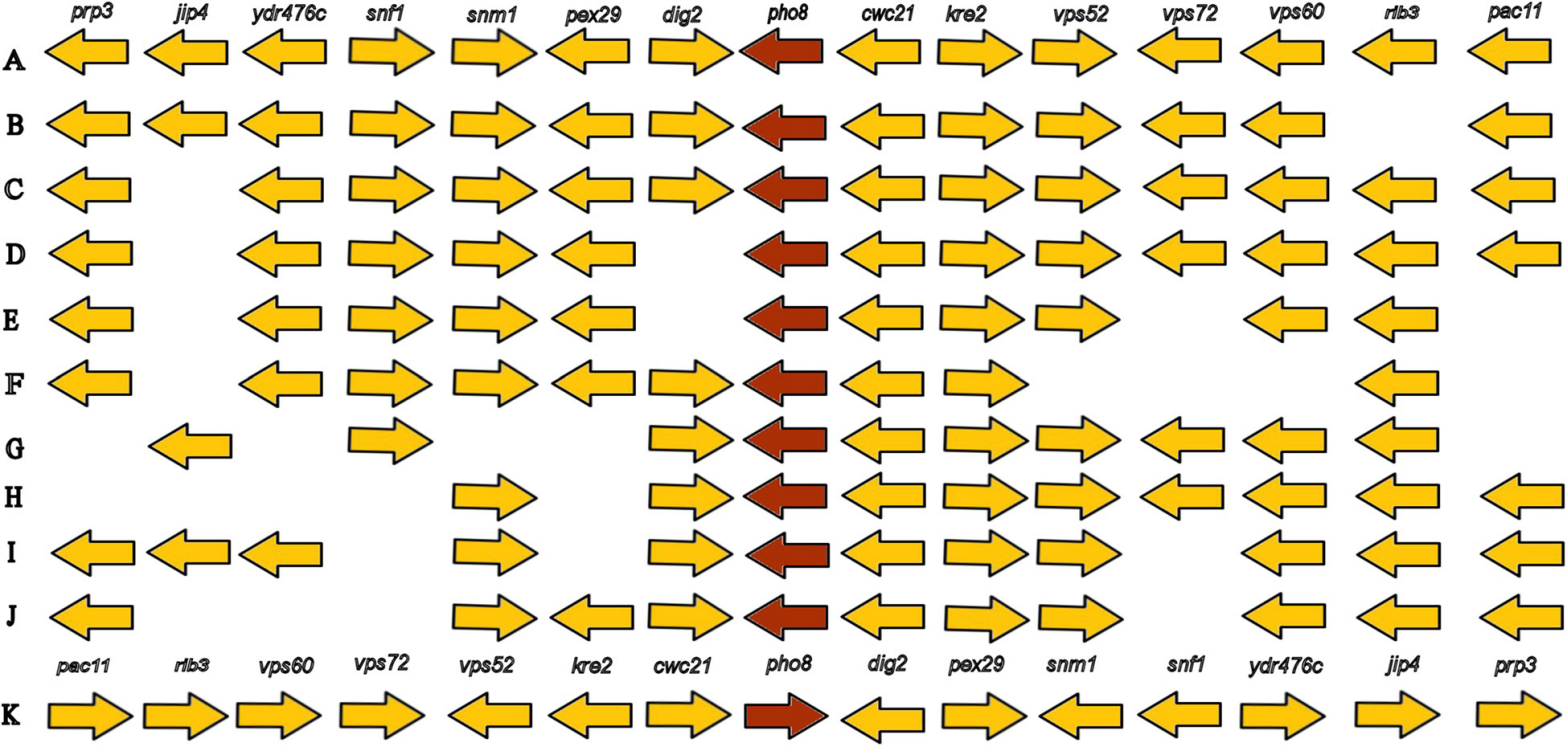
**Genomic neighborhood of the 63 kDa phosphatase (PHO8) coding gene: (A) *Sb *EDRL, and *S. cerevisiae *strains S288c, AWRI796, BY4741, BY4742, YJM789, Kyokai no. 7, EC1118, CEN.PK113-7D, FostersB, FostersO, JAY291, Lalvin QA23, Sigma1278b, T7, Vin13, VL3, W303, YJM789, ZTW1; (B) *S. cerevisiae *strain PW5; (C) *S. cerevisiae strain *EC9-8; (D) *S. cerevisiae strain *YJM269; (E) *S. cerevisiae strain *AWRI1631; (F) *S. cerevisiae strain *UC5; (G) *S. cerevisiae strain *YPS163; (H) *S. cerevisiae strain *FL100; (I) *S. cerevisiae strain *CBS7960; (J) *S. cerevisiae strain *CLIB215; (K) *S. cerevisiae strain *RM11-1a.** The genes mentioned are: *prp3* (YDR473C, Pre-mRNA processing); *jip4* (YDR475C, Jumonji domain interacting protein); YDR476C (Putative protein of unknown function; Green fluorescent protein (GFP)-fusion protein localizes to the endoplasmic reticulum); *snf1* (YDR477W, Sucrose non-fermenting); *snm1* (YDR478W, Suppressor of nuclear mitochondrial endoribonuclease 1); *pex2*9 (YDR479C, Peroxisomal integral membrane peroxin); *dig2* (YDR480W, Down-regulator of invasive growth); *pho8p* (YDR481C, Phosphate metabolism); *cwc21* (YDR482C, Complexed with Cef1p 1); *kre2* (YDR483W, Killer toxin resistant); vps52 (YDR484W, Vacuolar protein sorting); *vps72* (YDR485C, Vacuolar protein sorting 1); *vps60* (YDR486C, Vacuolar protein sorting); *rib3* (YDR487C, Riboflavin biosynthesis); *pac11* (YDR488C, Perish in the absence of CIN8 1).

A 63-kDa phosphatase of *Sb* (PHO8P) containing a peptide of 38-amino acids (HFIGSSRTRSSDSLVTDSAAGATAFACALKSYNGAIGV), has been suggested to inhibit the toxicity of *E. coli* surface endotoxins by dephosphorylation [18]. The sequence has an activation site (VTDSAAGAT) that is present in *S. cerevisiae* and *S. pombe*. The presence of this protein was examined in most of the *S. cerevisiae* genomes available at the *Saccharomyces* genome database (SGD) [42] and in our *Sb* genome. *S. cerevisiae* strains AWRI1631, AWRI796, BY4741, BY4742, CBS7960, CEN.PK113-7D, CLIB215, CLIB324, CLIB382, EC1118, EC9-8, FL100, FostersB, FostersO, JAY291, Kyokai no.7, Lalvin QA23, M22, PW5, RM11-1a, Sigma1278b, S288c, T73, T7, UC5, Vin13, VL3, W303, Y10, YJM269, YJM789, YPS163 and ZTW1 were considered, out of which the phosphatase PHO8P was present in 28 strains and could not be found in strains CLIB324, T73, Y10, CLIB382 and M22. The *pho8* gene is present in the *Sb* genome reported here. Fourteen genes (*prp3*, *jip4*, YDR476C, *snf1*, *snm1*, *pex29*, *dig2*, *cwc21*, *kre2*, *vps52*, *vps72*, *vps60*, *rib3*, *pac11*) that are in the genomic neighborhood of the phosphatase gene were analyzed. All these genes are completely conserved in *Sb,* S288c, AWRI796, BY4741, BY4742, CEN.PK113-7D, EC1118, FostersB, FostersO, JAY291, Kyokai no. 7, Lalvin QA23, Sigma1278b, T7, Vin13, VL3, W303, YJM789 and ZTW1 strains of *S. cerevisiae* (Figure 2A). The *S. cerevisiae* RM11-1a (Figure 2K) has likewise all genes conserved but they are present on the opposite strand. Other *S. cerevisiae* strains have one or more of these 14 genes missing (Figure 2B-J). The protein was conserved among all the *S. cerevisiae* strains with >95% identity suggesting the possibility of phosphatase activity in all the *S. cerevisiae* strains.

### Galactose metabolism

**Figure 3:**
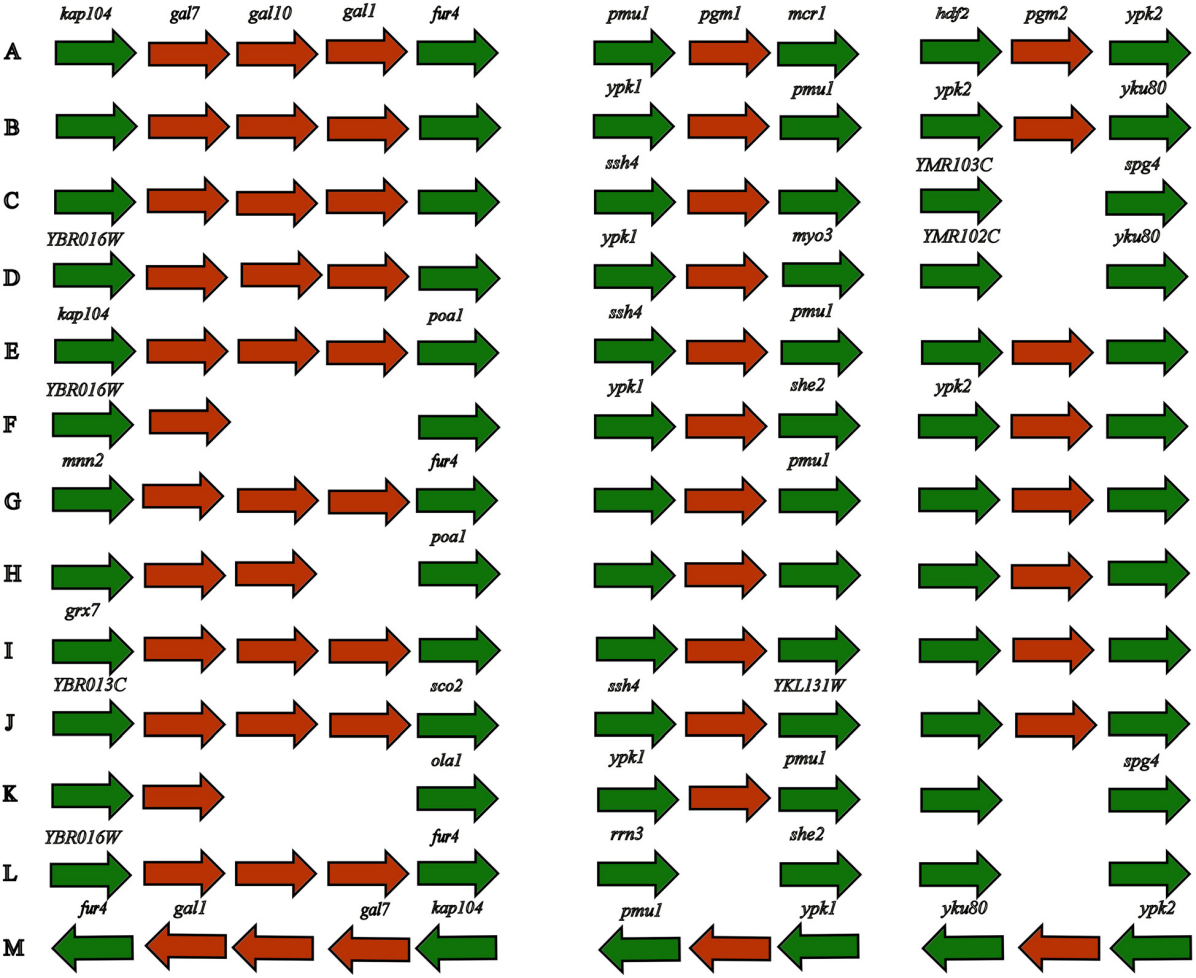
**Leloir pathway genes for galactose metabolism: Genomic neighborhood of genes involved in galactose metabolism. (A) ***Sb* EDRL; **(B) ***S. cerevisiae strains* S288c, AWRI1631, AWRI796, BY4741, BY4742, CEN.PK113-7D, EC1118, FostersB, FostersO, JAY291, Kyokai no. 7, Lalvin QA23, Sigma1278b, T7, UC5, Vin13, VL3, YJM789, W303, ZTW1; **(C) ***S. cerevisiae strain* FL100; **(D) ***S. cerevisiae strain* EC9-8; **(E) ***S. cerevisiae strain* PW5; **(F )***S. cerevisiae strain* CLIB324; **(G) ***S. cerevisiae strain* YJM269; **(H) ***S. cerevisiae strain* CLIB215; **(I) ***S. cerevisiae strain* CBS7960; **(J) ***S. cerevisiae strain* M22; **(K) ***S. cerevisiae strain* YPS163; **(L) ***S. cerevisiae strain* T73; **(M) ***S. cerevisiae strain* RM11-1A. The genes shown are: YBR013C (Uncharacterized ORF); *grx7* (YBR014C, Glutaredoxin 2); *mnn2* (YBR015C, Mannosyltransferase); YBR016W; *kap104* (YBR017C, Karyopherin); *gal1* (YBR020W, Galactose metabolism); *gal7* (YBR018C, Galactose metabolism); *gal10* (YBR019C, Galactose metabolism); *fur4* (YBR021W, 5-Fluorouridine sensitivity); *poa1* (YBR022W, Phosphatase Of ADP-ribose 1’-phosphate); *chs3* (YBR023C, Chitin Synthase); *sco2* (YBR024C, Suppressor of Cytochrome oxidase deficiency); *ola1* (YBR025C, Obg-like ATPase); *rma1* (YKL132C, Reduced Mating A); *she2* (YKL130C, Swi5p-dependent HO Expression); *myo3* (YKL129C, Myosin); *pmu1* (YKL128C, Phosphomutase); *pgm1* (YKL127W, Phosphoglucomutase); *ypk1* (YKL126W, Yeast protein kinase); *rrn3* (YKL125W, Regulation of RNA Polymerase); *ssh4* (YKL124W, Suppressor of SHr3 deletion); *srt1* (YMR101C, Cis-prenyltransferase); YMR102C; YMR103C; *ypk2* (YMR104C, Yeast protein kinase); *pgm2* (YMR105C, Phosphoglucomutase); *yku80* (YMR106C, Yeast KU protein); *spg4* (YMR107C, Stationary Phase gene).

One of the distinguishing features of *Sb* is that unlike *S. cerevisiae*, it is incapable of utilizing galactose as a source of carbon [23,28,29,40,43]. However there are reports of some *Sb* strains, which can utilize galactose [29]. The enzymes galactose-mutarotase, galactokinase, galactose-1-phosphate uridyltransferase, UDP-galactose-4-epimerase and phosphoglucomutase are constituents of the Leloir pathway which helps to catalyze the conversion of galactose to glucose-6-phosphate [29]. Glucose-6-phosphate can then further be utilized via the glycolysis pathway. Galactokinase, galactose-1-phosphate uridyltransferase and UDP-galactose-4-epimerase are in synteny in most of the *S. cerevisiae* strains such as RM11-1a, S288c, AWRI796, BY4741, BY4742, Fosters B, FostersO, JAY291, LalvinQA23, Sigma1278b, T7, UC5, Vin13, VL3, YJM789, AWRI1631, CEN.PK113-7D, EC1118, Kyokai no. 7, ZTW1 and a similar synteny was observed for our *Sb* (Figure 3). *S. cerevisiae* strains CLIB382 and Y10 were not included in this analysis as their genomes are not annotated completely on the SGD. Surprisingly, our *Sb* genome has all the genes responsible for galactose uptake and fermentation, although it has been suggested that *Sb* is not able to utilize galactose as a carbon source [28,44]. We have experimentally confirmed that *Sb* EDRL whose genome is reported here can assimilate galactose but cannot ferment it (unpublished results). Given that all genes responsible for galactose assimilation and fermentation are present in our genome, an effect similar to that observed by van den Brink *et. al.,* 2009 [45] for *S. cerevisiae* CEN.PK113-7D wherein the yeast is unable to suddenly switch from glucose to galactose in the absence of oxygen (fermentation), due to energetic requirements, may help explain the inability of our *Sb* to ferment galactose.

### Future directions

The genome of *Sb* offers an important starting point to glean insights into the probiotic effects of this yeast and help differentiate it from other strains of *S. cerevisiae.* It is possible that the various probiotic effects that have been attributed to *Sb* do not necessarily correspond to the same strain of this organism. Therefore it will be of interest to obtain genomes of different commercially available strains of *Sb* in order to get a complete genome-level understanding of this important probiotic yeast.

## Conclusions

The first draft genome of *Sb* provides a framework to understand at a molecular level, some of the properties of this novel probiotic yeast. While it has been shown that *Sb* strains do not utilize galactose [28], our genome surprisingly reveals the conservation of all enzymes involved in the Leloir pathway. In addition, we were able to locate a 63 kDa phosphatase that has been suggested to inhibit the toxicity of *E. coli* surface endotoxins [17]. Furthermore, we could shortlist putative candidates for the 54 kDa serine protease and the 120 kDa protein that confers additional probiotic functions to *Sb*. It is of interest to note that none of these proteins however are unique to *Sb* and have close homologues in other strains of *S. cerevisiae*.

### Availability of supporting data

This Whole Genome Shotgun project has been deposited at DDBJ/EMBL/GenBank under the accession ATCS00000000 for *Saccharomyces cerevisiae* strain *boulardii* EDRL. The version described in this paper is the first version ATCS01000000.

## Abbreviations

Sb: *Saccharomyces boulardii*; GIT: Gastrointestinal tract; GI: Gastrointestinal; PCR: Polymerase chain reaction; RFLP: Restriction fragment length polymorphism; PFGE: Pulsed field gel electrophoresis; PMS: Post-meiotic segregation; CDS: Coding sequence; HKY: Hasegawa, Kishino and Yano; OD: Optical density; cAMP: Cyclic adenosine monophosphate; EHEC: Enterohemorrhagic *Escherichia coli*; COX2: Cytochrome-c oxidase II; MLSA: Multi locus sequence analysis; FFAS: Fold and function assignment system; PCMA: Profile consistency multiple sequence alignment; ITS: Internal transcribed spacer; MAPK: Mitogen-activated protein kinase; PERL: Practical extraction and report language; GO: Gene ontology; STRING: Search tool for the retrieval of interacting genes/proteins; YM: Yeast mold; YEPD: Yeast extract-peptone-dextrose; C-CAMP: Centre for cellular and molecular platforms; EDRL: Econorm - Dr. Reddy’s Laboratories; SGD: *Saccharomyces* genome database.

## Competing interest

The authors declare that they have no competing interests.

## Authors’ contributions

SS and RTNC conceived the idea; AA isolated genomic DNA and carried out strain identification and ITS sequencing; RT and GSP carried out microsatellite analysis. IK, KK and SS carried out the quality control, assembly, annotation and analysis of the genomic data. IK, AA, RTNC and SS wrote the manuscript. All authors have read and approved the manuscript.

## Supplementary Material

Additional file 1 Reference-based mapping assembly report (sorted on basis of percentage of reads mapped). Click here for file

Additional file 2 The genomic positions of putative 120 kDa proteins, 54 kDa serine proteases, 63 kDa phosphatase with its genomic neighbors and proteins involved in galactose metabolism. Click here for file

Additional file 3 Protein sequences of putative 120 kDa proteins, 54 kDa serine proteases, 63 kDa phosphatase with its genomic neighbors and proteins involved in galactose metabolism. Click here for file
